# Supplementary material for: Probiotic Supplementation in Chronic Kidney Disease: Outcomes on Uremic Toxins, Inflammation, and Vascular Calcification from Experimental and Clinical Models
Source: Toxins (Basel). 2025 Dec 20;18(1):6. doi: 10.3390/toxins18010006 (PMC12846724; doi:10.3390/toxins18010006)
Supplement: Supplementary file 1 [file toxins-18-00006-s001.zip › toxins-3963422-supplementary.pdf]

toxins

MDPI

Supplementary Materials: Probiotic Supplementation in Chronic Kidney Disease: Outcomes on Uremic Toxins, Inflammation, and Vascular Calcification from Experimental and Clinical Models

Table S1. Inflammatory panel. 92 proteins measured by PEA essay.

Abbreviation	Name	Abbreviation	Name
**4E-BP1**	Eukaryotic Translation Initiation Factor 4E-Binding Protein 1	**IL8**	Interleukin 8
**ADA**	Adenosine Deaminase	**IL10**	Interleukin 10
**ARTN**	Artemin	**IL-10RA**	Interleukin 10 Receptor Subunit Alpha
**AXIN1**	Axin 1	**IL-10RB**	Interleukin 10 Receptor Subunit Beta
**β-NGF**	Beta Nerve Growth Factor	**IL12B**	Interleukin 12 Subunit Beta
**CASP-8**	Caspase-8	**IL13**	Interleukin 13
**CCL3**	C-C Motif Chemokine Ligand 3	**IL-15RA**	Interleukin-15 receptor subunitalpha
**CCL4**	C-C Motif Chemokine Ligand 4	**IL17A**	Interleukin 17A
**CCL11**	C-C Motif Chemokine Ligand 11	**IL-17C**	Interleukin 17C
**CCL19**	C-C Motif Chemokine Ligand 19	**IL18**	Interleukin 18
**CCL20**	C-C Motif Chemokine Ligand 20	**IL-18R1**	Interleukin 18 Receptor 1
**CCL23**	C-C Motif Chemokine Ligand 23	**IL20**	Interleukin 20
**CCL25**	C-C Motif Chemokine Ligand 25	**IL-20RA**	Interleukin 20 Receptor Subunit Alpha
**CCL28**	C-C Motif Chemokine Ligand 28	**IL-22 RA1**	Interleukin-22 receptor subunit alpha-1
**CD244**	Natural Killer Cell Receptor 2B4	**IL24**	Interleukin 24
**CD5**	CD5 Molecule	**IL33**	Interleukin 33
**CD6**	CD6 Molecule	**LAP TGF-β-1**	Latency-Associated Peptide of Transforming Growth Factor Beta 1
**CD8A**	CD8a Molecule	**LIF**	Leukemia Inhibitory Factor
**CD40**	CD40 Molecule	**LIF-R**	Leukemia Inhibitory Factor Receptor
**CDCP1**	CUB Domain-Containing Protein 1	**MCP-1**	Monocyte Chemoattractant Protein 1
**CSF-1**	Colony Stimulating Factor 1	**MCP-2**	Monocyte Chemoattractant Protein 2
**CST5**	Cystatin D	**MCP-3**	Monocyte Chemoattractant Protein 3
**CX3CL1**	Fractalkine	**MCP-4**	Monocyte Chemoattractant Protein 4
**CXCL1**	C-X-C Motif Chemokine Ligand 1	**MMP-1**	Interstitialcollagenase
**CXCL5**	C-X-C Motif Chemokine Ligand 5	**MMP-10**	Stromelysin-2
**CXCL6**	C-X-C Motif Chemokine Ligand 6	**NRTN**	Neurturin
**CXCL9**	C-X-C Motif Chemokine Ligand 9	**NT-3**	Neurotrophin-3
**CXCL10**	C-X-C Motif Chemokine Ligand 10	**OPG**	Osteoprotegerin
**CXCL11**	C-X-C Motif Chemokine Ligand 11	**OSM**	Oncostatin M
**DNER**	Delta and Notch-like epidermal growth factor-related receptor	**PD-L1**	Programmed Death-Ligand 1
**EN-RAGE**	Extracellular Newly identified RAGE-binding protein	**SCF**	Stem Cell Factor
**FGF-5**	Fibroblast Growth Factor 5	**SIRT2**	Sirtuin 2
**FGF-19**	Fibroblast Growth Factor 19	**SLAMF1**	SLAM Family Member 1
**FGF-21**	Fibroblast Growth Factor 21	**ST1A1**	Sulfotransferase Family 1A Member 1

**FGF-23**	Fibroblast Growth Factor 23	**STAMBP**	Signal Transducing Adaptor Molecule Binding Protein
**Flt3L**	Fms-related Tyrosine Kinase 3 Ligand	**TGF-α**	Transforming Growth Factor Alpha
**GDNF**	Glial Cell Line-Derived Neurotrophic Factor	**TNF**	Tumor Necrosis Factor
**HGF**	Hepatocyte Growth Factor	**TNFB**	Tumor Necrosis Factor Beta
**IFN-γ**	Interferon Gamma	**TNFRSF9**	Tumor Necrosis Factor Receptor Superfamily Member 9
**IL1-α**	Interleukin 1 Alpha	**TNFSF14**	Tumor Necrosis Factor Superfamily Member 14
**IL2**	Interleukin 2	**TRAIL**	TNF-Related Apoptosis-Inducing Ligand
**IL-2RB**	Interleukin-2 receptor subunit beta	**TRANCE**	TNF-Related Activation-Induced Cytokine
**IL4**	Interleukin 4	**TSLP**	Thymic Stromal Lymphopoietin
**IL5**	Interleukin 5	**TWEAK**	TNF-Related Weak Inducer of Apoptosis
**IL6**	Interleukin 6	**uPA**	Urokinase-type Plasminogen Activator
**IL7**	Interleukin 7	**VEGFA**	Vascular Endothelial Growth Factor A

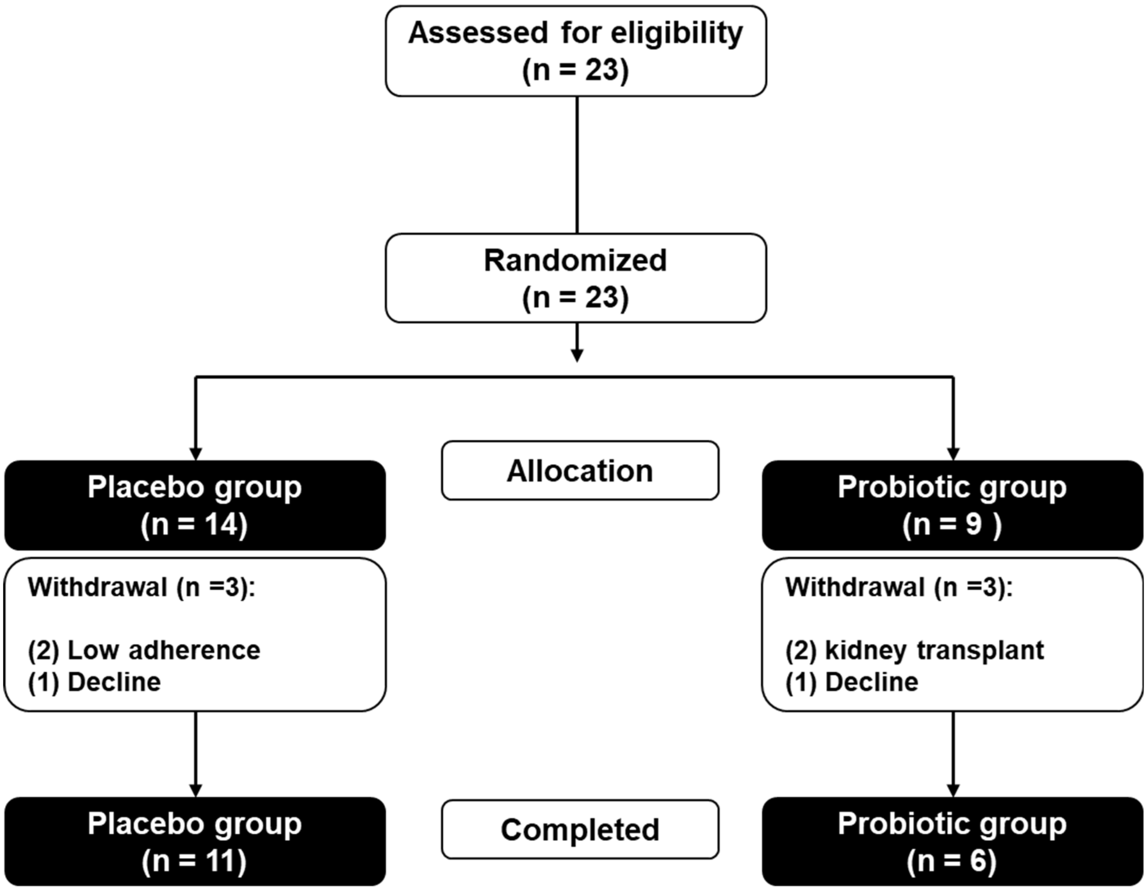

Supplementary Figure S1. Clinical trial flowchart.

Supplementary material

Inclusion criteria were patients with CKD stages 4 or 5 and with evidence of VC assessed by X-ray or computed tomography (CT). Dialysis vintage was of at least one month before entering the study. Exclusion criteria were as follows: patients diagnosed or suspected of having inflammatory bowel disease, treated with antibiotics during the 2 months before starting the study, recurrent infections or diverticula treated with antibiotics, HIV infection, hepatitis B or C, chronic liver disease, recombinant or immunosuppressive therapies, women who are breastfeeding, pregnant or intend to be, or previous history of cancer in the last 5 years.

Randomization was performed automatically using the RedCap data collection system (Research Electronic Data Capture), aiming to homogenize the groups based on sex, age, glomerular filtration rate and albuminuria. In addition, all data were also collected in REDCap.
